# Supplementary material for: Identification of photoperiod-induced specific miRNAs in the adrenal glands of Sunite sheep (Ovis aries)
Source: Front Vet Sci. 2022 Jul 22;9:888207. doi: 10.3389/fvets.2022.888207 (PMC9354845; doi:10.3389/fvets.2022.888207)
Supplement: Supplementary file 1 [file Data_Sheet_1.ZIP › Supplementary file/miRDeep2 file.zip!/miRDeep2 file/Know_Structure/SP42A3/oar-mir-218a.pdf]

miRBase precursor	: oar-mir-218a
Total read count	: 40375
oar-miR-218a read count	: 40375
remaining reads	: 0

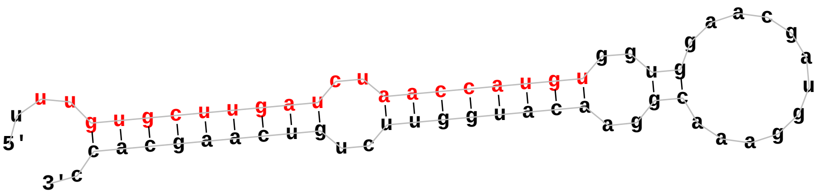

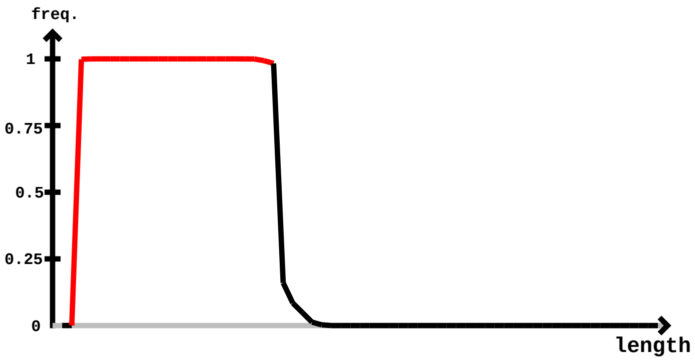

oar-miR-218a

```
5'- uuugugcuugaucuaaccaugugguggaacgauggaaacggaacaugguucugucaagcacc   -3'    exp
    ...(((((((((..((((((((..((............))..))))))))..))))))))).    reads      mm         sample
    uuugugcuugaucuaaccaug.......................................     1          0          seq
    uuugugcuugaucuaaccaugu......................................     7          0          seq
    uuugugcuugaucuaaccaugug.....................................     1          0          seq
    .uugugcuugaucuaacc..........................................     7          0          seq
    .uugAgcuugaucuaacca.........................................     2          1          seq
    .uugugcuugaucuaacca.........................................     8          0          seq
    .uugugcuugaucuaaccau........................................     254        0          seq
    .uugugcuugaucuaaccaug.......................................     411        0          seq
    .uugugcuugaucuaacAaugu......................................     98         1          seq
    .uugugcuugaucuaaccaugu......................................     33110      0          seq
    .uugugcuugaucuaaccaugug.....................................     3052       0          seq
    .uugugcuugaucuaaccaugugg....................................     1417       0          seq
    .uugugcuugaucuaaccauguggu...................................     1438       0          seq
    .uugugcuugaucuaaccauguggug..................................     9          0          seq
    .uugugcuugaucuaaccauguggU...................................     385        1          seq
    .uugugcuugaucuaaccauguggUg..................................     117        1          seq
    ..ugugcuugaucuaaccaugu......................................     13         0          seq
    ..ugugcuugaucuaaccaugug.....................................     2          0          seq
    ..ugugcuugaucuaaccaugugg....................................     22         0          seq
    ..ugugcuugaucuaaccauguggu...................................     11         0          seq
    ..ugugcuugaucuaaccauguggU...................................     3          1          seq
    ..ugugcuugaucuaaccauguggug..................................     1          0          seq
    ..ugugcuugaucuaaccauguggUg..................................     2          1          seq
    ...gugcuugaucuaaccaugu......................................     1          0          seq
    ...gugcuugaucuaaccaugug.....................................     1          0          seq
    ....ugcuugaucuaaccauguggu...................................     1          0          seq
    ....ugcuugaucuaaccauguggU...................................     1          1          seq
```
